# Permeation through the Cell Membrane of a Boron-Based β-Lactamase Inhibitor

**DOI:** 10.1371/journal.pone.0023187

**Published:** 2011-08-17

**Authors:** Manuela Minozzi, Gianluca Lattanzi, Roland Benz, Maria P. Costi, Alberto Venturelli, Paolo Carloni

**Affiliations:** 1 Modeling & Simulation Lab Department of Studies on Structures, University “Roma Tre”, Roma, Italy; 2 Department of Basic Medical Sciences, TIRES Centre and Istituto Nazionale di Fisica Nucleare, University of Bari “Aldo Moro”, Bari, Italy; 3 School of Engineering and Science Jacobs-University Bremen, Bremen, Germany; 4 University of Modena and Reggio Emilia, Pharmaceutical Sciences Dept., Modena, Italy; 5 German Research School for Simulation Science, Jülich Research Center and RWTH-Aachen University, Jülich, Germany; University of Cambridge, United Kingdom

## Abstract

Bacteria express beta-lactamases to counteract the beneficial action of antibiotics. Benzo[b]-thiophene-2-boronic acid (BZB) derivatives are β-lactamase inhibitors and, as such, promising compounds to be associated with β-lactam antibacterial therapies. The uncharged form of BZB, in particular, is suggested to diffuse through the outer membrane of Gram negative bacteria. In this study, through the combination of electrophysiological experiments across reconstituted PC/n-decane bilayers and metadynamics-based free energy calculations, we investigate the permeation mechanism of boronic compounds. Our experimental data establish that BZB passes through the membrane, while computer simulations provide hints for the existence of an aqueous, water-filled monomolecular channel. These findings provide new perspectives for the design of boronic acid derivatives with high membrane permeability.

## Introduction

**Figure 1:**
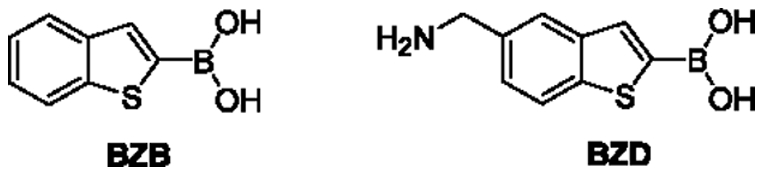
βLs boronic inhibitors. BZB benzo[b]thiophene-2-boronic acid, BZD 5-aminomethyl benzo[b]thiophene-2-boronic acid.

The onset of Gram-negative bacteria's resistance to β-lactam antibiotics is a major threat to public health [1]. The widespread use of this compound class caused the development of resistance mechanisms that make these drugs ineffective. There are different resistance mechanisms to counteract the activity of β-lactam antibiotics. One of them is the expression of β-lactamase (βLs), enzymes that catalyze the hydrolysis of the β-lactam ring of the antibiotic, destroying hereby their antibacterial activity [2], [3], [4]. Inhibitors structurally similar to these antibiotics, featuring the β-lactam ring, have been developed to block the βLs action. The broad activity of class C β-Ls and the regulatory response to classic β-lactams motivated the search for novel inhibitors structurally unrelated to β-lactams. Non-β-lactam inhibitors are able to evade pre-evolved bacterial resistance mechanism: they are not recognized by β-lactam signalling proteins, are not affected by porin channel mutations responsible for decreasing permeability, and, lacking the β-lactam core, they should not be hydrolyzed by mutant enzymes that arise in response to new β-lactams [5], [6], [7], [8]. Among those, benzo[b]-thiophene-2-boronic acid (BZB, Fig. 1) is one of the highest potent β-lactamase inhibitor boronic compounds in vitro (K_i_ = 27 nM towards ampicillin resistant class C β-lactamase, AmpC) [8], [9], [10], [11], [12], [13]. Despite its tight binding and ligand efficacy, BZB showed only modest celluar activity and when administered in combination with third generation cephalosporins like ceftazidime (CAZ*a*), it was only active in the tens-of-micromolar range in antimicrobial cell-based assays, a thousand-fold worse than its K_i_ value [10], [14], [15], [16]. Such low in vivo efficiency is likely to be related to inefficient membrane permeation. Experiments in which polymixine was used to disaggregate the membrane showed an higher amount of compound entering the cells, inducing significant minimum inhibitory concentration amelioration: the efficiency observed was then closer to the effective Ki versus the enzyme. More recently, Venturelli et al. have identified 5-aminomethylbenzo[*b*]thiophen-2-boronic acid (BZD) as a BZB's derivative with an improved permeability index (PI) [10] and better cell efficacy despite its higher K_i_ (260 nM) [10], [14].

**Figure 2:**
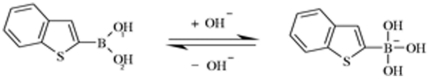
BZB reversible adducts formed with water hydroxyl, resembling the high energy intermediate of enzyme reaction.

Our previous studies on BZD [15], [16] focused on its passage through the outer membrane via porin channels, the same route supposed for β-lactams themselves. At variance, BZB is supposed to diffuse passively through the outer membrane: for this series of inhibitors, structural variations strongly influence the route to cell entry. The low efficiency of BZB is caused most probably by an excess of the negatively charged form (about 90%) due to the pK_a_ value of the boronic group (pKa = 6.39) at physiological pH [17]. The negatively charged form is expected to cross the membrane with very low efficiency, since the membrane is lipophilic. The less abundant neutral form is expected to pass more efficiently and is probably responsible for the antimicrobial activity as observed for other β-lactam antibiotics [17]. This effect has never been studied for the boronic compound class. A deeper investigation of the permeation process aimed at understanding how structural features of compounds may influence membrane crossing, may provide useful hints to the design of novel boron-based drugs with improved permeability efficiency [18], [19]. Here we address this issue through a combination of electrophysiological experiments and atomistic simulations. Experiments with reconstituted membranes, made of PC/n-decane, were carried out using BZB and BZD for comparison in the presence or absence of OmpF porins, at different pH values. The dependence of the electrophysiological behavior on pH is consistent with the fact that the percentage of the neutral and negatively charged forms (Fig. 2) changes significantly. In particular, the negative form passes from 90% at pH = 7.35 to 29% at pH = 6.

Electrophysiological experiments were carried out on BZD that, differently from BZB, was expected to cross the membrane through membrane porins that are permeable to cationic antibiotics. The pKa of the boronic group is the same as for BZB (pKa = 6.39) [14] while the amino group (pKa = 9.26) [14] is positively charged at physiological pH, therefore it represents the optimal compound for comparison with BZB in our experimental conditions.

While a model of the membrane translocation of negatively charged antibiotics and low water soluble compounds has already been proposed [20], the model for the translocation of boronic acid derivatives across bacterial membranes is still a matter of debate. Here, we present a model that is consistent with the experimental data, by performing atomistic molecular dynamics simulations to investigate the permeation of BZB through the bacterial membrane, modeled as a POPC bilayer. Since the transport mechanism is very likely to be associated with a high activation barrier, we used the metadynamics method [21], [22] to evaluate the free energy profile for the translocation of the compound through the membrane. This technique has been widely tested and used in a variety of biophysical applications [22], including permeation of antibiotics through porins [23], [24], [25].

## Results and Discussion

To establish the membrane permeation mechanism of the BZB at physiological pH (pH = 7.35), our investigation proceeded in several steps. First, we used electrophysiological methods to assess whether BZB passes through the membrane, through membrane porins or through both and which form of BZB, negatively charged or neutral, could cross the membrane. Then, we used metadynamics simulations to investigate the molecular determinants of the permeation process.

We measured the single-channel conductance (SCC) of lipid bilayer membranes made of PC/n-decane in the presence of OmpF porins, in unbuffered 1 M KCl (pH is about 6) with or without BZB. At this pH, BZB is present as 71% in neutral form and 29% in negatively charged form. The negative form of BZB cannot pass through OmpF porins because these proteins are selective for cations and tend to block also in vivo transport of negatively charged bile acids into the bacteria [26]. On the other hand, OmpF porins are known to let hydrophilic antibiotics pass [7], [27]. If BZB permeates, at least in part, through the porins, the SCC must decrease upon addition of BZB [28], [29]. In our experiments the SCC of the same system plus 0.5 mM BZB on both sides of the membrane was 4.1 nS, very similar to the SCC of the membrane alone [26], [30] (see Text S1 for details). The same result was also obtained with a larger number of OmpF pores reconstituted into the membranes (OmpF concentration 20 ng/mL) and with further additions of 0.15 mM BZB on both sides of the membrane (increasing BZB concentration from 1 to 2.9 mM). The results for single- and multi-channel experiments thus clearly indicate that BZB translocation does not depend on porins and is a process that takes place exclusively through the membrane.

**Figure 3:**
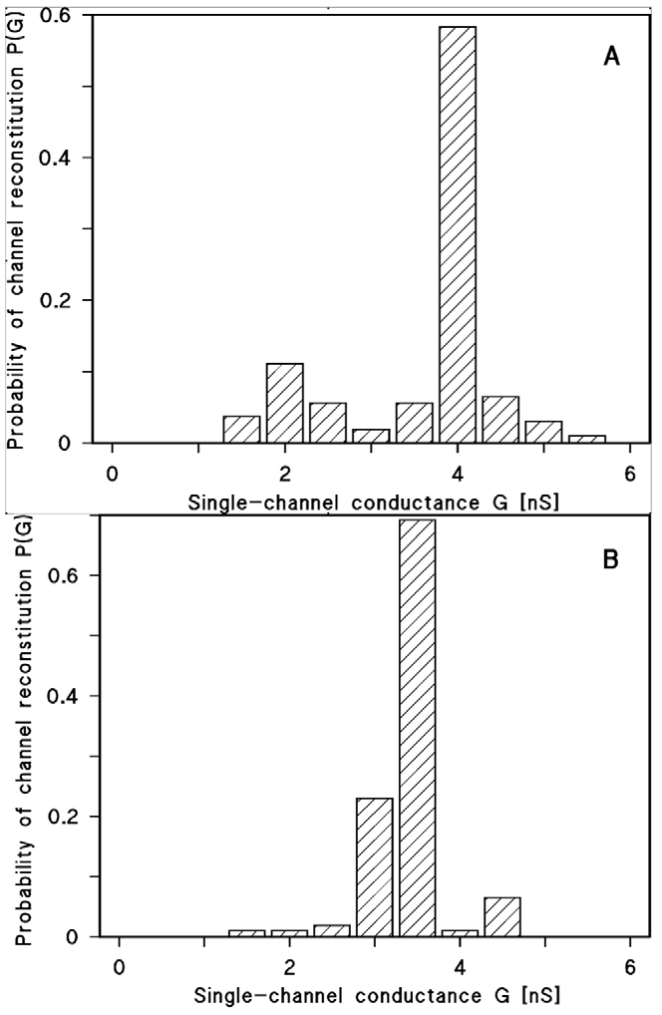
Histogram of the probability P(G) for the occurrence of a given conductivity unit. Probability observed with lipid bilayer membranes in the presence of OmpF alone (A) or when the aqueous phase contained in addition 0.45 mM BZD (B). P(G) is the probability that a given conductance increment G is observed in the single-channel experiments calculated as reported in Fig. S1.

Similar experiments were also performed with BZD. Interestingly, we observed in single-channel experiments a small but significant decrease of conductance presumably because the bulky BZD could enter the porin channel thus hindering the flux of ions through the channel. Figure 3 shows histograms of the single channel conductance distributions in absence (Fig. 3A) and in presence of BZD (Fig. 3B). The single channel conductance of OmpF decreased from an average 4.1 nS (Fig. 3A) to 3.4 nS (Fig. 3B) when 0.45 mM BZD was added to the aqueous phase. Similar effects on porin conductance have also been observed in previous studies with other compounds including antibiotics [28], [29].

In subsequent experiments (data not shown), a large number of OmpF pores were reconstituted into lipid bilayer membranes. Then BZD was added to the aqueous phase on both sides of the membrane in increasing concentrations starting from 0.15 mM. The addition of BZD resulted in a further decrease of membrane conductance caused by the same effect as described above for the single-channel measurements. Hence we conclude that BZD is able to enter the OmpF pores and to block in part the current through the OmpF channels.

**Figure 4:**
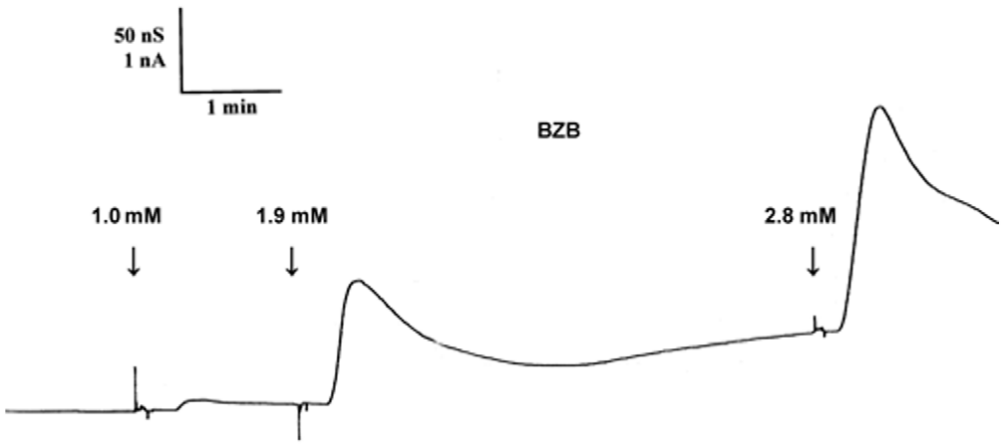
Recording of PC/n-decane membrane conductance measured at pH 7.35 after addition of BZB in increasing concentrations from 1.0 mM to 2.9 mM.

In a second step, we investigated the permeation of BZB through a PC/n-decane membrane. We measured the membrane conductance at physiological pH (pH 7.35) in which 90% of BZB is present in its negative form and only 10% in its neutral form. When increasing concentrations of BZB were added to both sides of the membrane starting from 0.15 mM up to 2.9 mM, we observed transient increases of membrane conductance following each BZB addition (see Fig. 4).

The current through unmodified lipid bilayer membranes is normally very low because these membranes have a resistance of about 100 GΩ (diameter 0.5 mm) in the absence of membrane-active substances. The addition of the charged BZB compounds increased the conductance of the membrane (current transient) because the compound acts like a lipophilic ion due to charge delocalisation of the negative charge in the benzothiazole ring. Lipophilic ions move through the membrane [17] with low efficiency and hence very slowly in comparison to neutral compounds. The current transient is caused by slow aqueous diffusion of the negatively charged BZB compound that moves faster through the membrane than through the aqueous phase (unstirred layers) at the membrane-water interface causing diffusion polarisation [17]. The neutral compound contributed to this process. Polar compounds tend to decrease the dipole potential of membranes when they are adsorbed in a direction that is perpendicular to the existing dipole potential. A typical such molecule is phloretin [31]. However this effect is difficult to measure. Although we conclude that both the negative and neutral forms of BZB pass through the lipid bilayer membranes, the neutral, more hydrophobic, form moves faster: as a consequence this form is transported through the membrane more efficiently and is therefore responsible for the biological activity, that is low given the low fraction of neutral form present.

**Figure 5:**
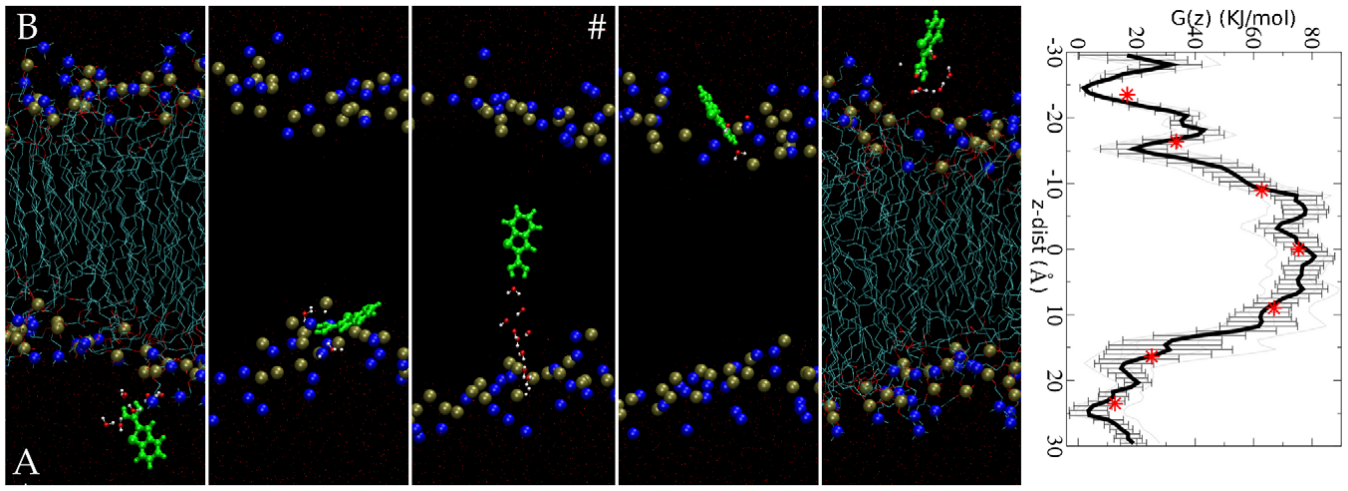
(Left) Representative snapshots of BZB permeation as obtained by metadynamics. The water channel accompanying BZB translocation is shown in ball and sticks at selected positions of the drug. The P and N atoms of POPC are displayed as orange and blue spheres, respectively. The whole POPC molecules are represented as lines in the first and last frame. (Right) G(z) calculated as a function of z-dist. G(z) is obtained as the average of 5 independent profiles. The mean value of the averaged FES within the four-region scheme is represented by red stars (see Text S1 for more details).

Since the neutral form is responsible for the biological activity and permeates through the membrane, we focused our computational studies on the translocation mechanism of this form. The free energy and the molecular mechanism of the process were reconstructed by metadynamics calculations [21], [22]. The resulting converged free energy profile *G(z)* is symmetric with respect to the central plane of the membrane, as expected (Fig. 5). It increases from the water phase into the hydrophobic core. The resulting activation free energy barrier is Δ*G* ^#^ = 63±8 KJ/mol. This result may be used to calculate the permeability coefficient (See Text S1 for details), which can be compared with the related experimental quantity. The latter has been measured for β-lactam antibiotics across the bacterial membrane [32] and for boric acids across membrane vesicles [33]. We calculated a value of permeability coefficient ranging from 7×10^−9^ to 8×10^−12^ cm/s. The upper value is in the range of experimental values measured with other systems [18]. Using the Arrhenius formula the barrier may also be associated to a timescale ranging from 10^−3^ s to 3 s. Further experiments are required to test the validity of these predictions.

**Figure 6:**
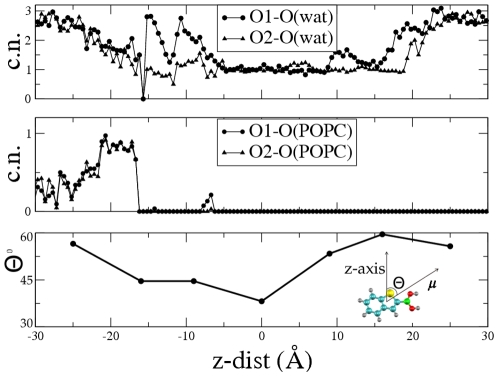
Dipole moment. Top: Coordination number of the O1 and O2 atoms of BZB with the water oxygens (O(wat), top) and POPC phosphate oxygens, (O(POPC), middle). Bottom: the angle Q between the dipole moment of the drug μ and the z-axis as a function of z-dist.

The inspection of the permeation mechanism clearly shows that the B-(OH)_2_ moiety H-bonds to one or more water molecules upon leaving the membrane surface: this is clearly shown by the B-(OH)_2_ –water coordination numbers (Fig. 6) as well as visual inspection of representative metadynamics snapshots (Fig. 5). The water molecule is connected to other water molecules in a chain-like monomolecular channel (Fig. 5). At the transition state, the channel connecting BZB with side A starts to break.

Drug permeation causes some rearrangement of the membrane surface. The calculated dipole of BZB (μ) is 2.85 Debye. It points towards the B-(OH)_2_ group (Fig. 6, bottom). The Θ angle between μ and the *z* direction is as small as 35° inside the membrane (Fig. 6, bottom) where the compound tends to align with the lipids tails. This has been observed for similar drugs [18], [34]. Instead Θ increases up to 60°, when the drug is in contact with the solvent, possibly because of the formation of H-bonds with the charged groups of the phospholipids as well as with water molecules. This is the molecular rationale for the observed behaviour that polar molecules tend to decrease the dipole potential of the membrane being absorbed in a direction that is perpendicular to the existing membrane dipole.

In this work, we have reported a combined experimental and computational study on the permeation of BZB through model membranes. Our experiments establish that BZB passes through the membrane both in charged and neutral form, as it was proposed in our previous work, where the neutral form, more lipophilic, is known to move faster (Fig. 1); the translocation of neutral BZB occurs via permeation though the membrane and is not assisted by porins. In our model the neutral BZB translocates assisted by a water channel bound to the boronic group. The neutral form is present in much smaller concentration than the negative one (about 10%) at pH 7.35. For comparison, the positively charged BZD compound (5-aminometil-BZB) with lower pKa, displays higher antibacterial activity and is shown to cross the membrane through porin channels. In this work, we have obtained more insights on the structural and energetic features associated with the permeation of BZB in the neutral form through the membrane via molecular dynamics simulations. Our calculations provide a permeability coefficient similar to that found for some antibiotics [32] and characterized by a translocation time ranging from 10^−3^ s to 3 s; they suggest that the hydrophilic part of the molecule is partially hydrated during the whole permeation process. In particular, a monomolecular water channel assists translocation, the BZB dipole tends to align to the lipid tails inside the membrane and, as a consequence, contribute to the overall SCC transient signal observed in our experiments.

This study provides mechanistic insight on how the efficient permeation of boronic derivatives affects antibacterial activity.

Medicinal chemistry usually adopts weak positively charged groups to increase the membrane permeability of candidate drugs that easily pass through the porins, as in the case of BZD and other positively charged derivatives. In this case, however, the option of a porin mutation is available and bacteria might develop a rapid resistance to these drugs. This resistance mechanism can be overcome by employing molecules that permeate directly through the bacterial membrane, as BZB derivatives. Unfortunately, however, membrane permeation can be slow and this decreases the antibacterial activity potential. Here we provide information on the structural determinants of BZB permeation through the membrane by molecular simulations. Our calculations show that a water-filled channel favors the membrane translocation. These observations could be used for chemical modifications of BZB to obtain compounds with improved membrane permeability.

## Materials and Methods

### Lipid bilayer experiments

BZB was provided by Sigma, BZD was synthesized using a previously published method [15], [16]. The methods used for the lipid bilayer experiments and the isolation of OmpF porin from Escherichia coli K12 have been described previously [26], [30]. In experiments with OmpF alone the membranes were formed from diphytanoyl phosphatidylcholine/n-decane and the aqueous phase contained 20 ng/ml of purified OmpF of *E. coli* K12 and 1 M KCl. The most frequent single channel conductance (about 58%) was 4.0 nS for 108 single channel events. The applied membrane potential was 20 mV; T = 20°C. The final data were calculated from measurements from 5 individual membranes. Experiments with BZD were conducted in the same conditions, but the aqueous phase contained in addition 0.45 mM BZD. Most frequent single-channel conductance was 3.5 nS for in total 104 single-channel events. The data were collected from more than 5 individual membranes. The aqueous phase contained 20 ng/ml OmpF, 1 M KCl and 0.45 mM BZD.

### Molecular Simulations

A 50 Å×50 Å POPC bilayer patch resulting in 60 1-palmitoyl-2-oleoyl-phosphatidylcholine (POPC) lipid molecules, was filled with TIP3P water molecules. The resulting system consisted of 16432 atoms. We reproduce some of the previously calculated properties, thus ensuring the validity of our parameterization (see Text S1). Then we added the BZB on the membrane surface, the parameters for the B(OH)_2_ moiety of BZB were taken from Tafi et al. [35] (see Text S1 for parameterization details.) Metadynamics calculations were performed with a locally modified version of NAMD2.6 [36]. Periodic boundary conditions were applied. Simulations were performed in an isothermal-isobaric ensemble (1 atm, 300 K). The metadynamics simulation was carried out after equilibration (see S.I). The free energy profile G(z) associated with BZB translocation through the lipid bilayer was calculated along the z-component of the distance vector joining the POPC membrane and the BZB center of mass (z-dist hereafter, Fig. 2). Following the suggestion of Laio et al. [37] we used the following parameters for metadynamics (details in Text S1): the time interval between the addition of two Gaussian functions τ = 100 fs, the Gaussian height w = 0.2 kJ/mol, the Gaussian width δ = 0.5 Å. The error on the reconstructed G(z) has been shown to be approximately determined by the ratio w/δτ [37]: our choice is consistent with ref. [24]. We inserted a rigid wall at z = ±33 Å to force the inhibitor inside the simulation box. The simulation was stopped after two complete cycles, i.e. two up-down and two down-up passages (15 ns). The reconstructed free energy profile converged after 11 ns (see Fig. S3a for details). The reconstructed G(z) was averaged over 5 free energy profiles after convergence, obtaining <G(z)> [38]. The lipids are highly anisotropic, therefore we also averaged the <G(z)> with respect to the z-dist coordinate inside a four regions scheme [39] (see Fig. S3). The permeability coefficient was calculated based on the estimated free energy barrier [39], [40] (see Text S1). Coordination numbers between BZB oxygens (O1,O2) (see Fig. 1) and water oxygens, (O(wat)), or the oxygens bound to the POPC phosphates, O(P-popc), were calculated counting the number of water oxygens and phosphate oxygens within a radius of 3.4 Å from O1 (O2). This is the distance between the first two peaks of the correlation function of water oxygens. The dipole moment was calculated based on the electrostatic term of the force field as in ref. [41].

## Supporting Information

Figure S1 **(A) Single-channel recording of a PC/n-decane membrane in presence of purified OmpF of E. coli K12.** About 10 min after the formation of the membrane, 20 ng/ml of OmpF were added to the aqueous phase on both sides of the membrane. The aqueous phase contained 1 M KCl. The applied membrane potential was 20 mV, and the temperature was 20°C. (B) Same conditions as in A, but the aqueous phase contained in addition 0.45 mM BZD. Note that the conductance of the single-channel steps in B were by about 14% smaller than those in A because of the interaction of the ion current through OmpF with BZD.
(TIF)

Figure S2 **Charge distribution of BZB** [**1**] **along with the dipole moment μ (D = 2.85 Debye).**
(TIF)

Figure S3 **a) Free energy profile calculated every 1 ns.** The potential landscape is gradually filled (dotted lines). Since the metadynamics converged (11 ns) we identified 5 different free energy profiles (black lines). b) G(z) as a function of z-dist (Å). The average (black line) of 5 independent profiles (Grey lines) is used to calculate the mean value inside the four region scheme (red starts). c) ball-and-sticks representation of a POPC molecule: P atoms are colored in blue, C6 atoms are colored in cyan, N atoms are colored in yellow. Bottom, right: snapshot of the equilibrated POPC bilayer. Vertical lines indicate the boundaries between the four regions defined in the text.
(TIF)

Text S1 (DOC)
