# Supplementary figures and images for: Permeation through the Cell Membrane of a Boron-Based β-Lactamase Inhibitor
Source: PLoS One. 2011 Aug 17;6(8):e23187. doi: 10.1371/journal.pone.0023187 (PMC3157353; doi:10.1371/journal.pone.0023187)

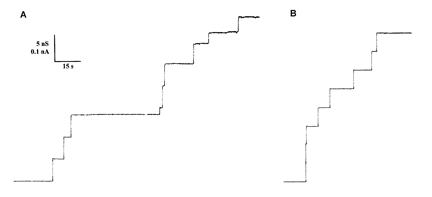

Supplement: Figure S1 — (A) Single-channel recording of a PC/n-decane membrane in presence of purified OmpF of E. coli K12. About 10 min after the formation of the membrane, 20 ng/ml of OmpF were added to the aqueous phase on both sides of the membrane. The aqueous phase contained 1 M KCl. The applied membrane potential was 20 mV, and the temperature was 20°C. (B) Same conditions as in A, but the aqueous phase contained in addition 0.45 mM BZD. Note that the conductance of the single-channel steps in B were by about 14% smaller than those in A because of the interaction of the ion current through OmpF with BZD. (TIF) [file pone.0023187.s001.tif]

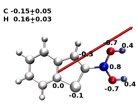

Supplement: Figure S2 — Charge distribution of BZB [1] along with the dipole moment μ (D = 2.85 Debye). (TIF) [file pone.0023187.s002.tif]

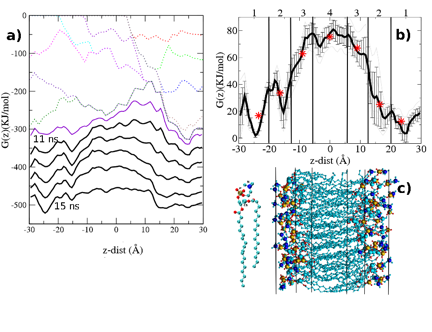

Supplement: Figure S3 — a) Free energy profile calculated every 1 ns. The potential landscape is gradually filled (dotted lines). Since the metadynamics converged (11 ns) we identified 5 different free energy profiles (black lines). b) G(z) as a function of z-dist (Å). The average (black line) of 5 independent profiles (Grey lines) is used to calculate the mean value inside the four region scheme (red starts). c) ball-and-sticks representation of a POPC molecule: P atoms are colored in blue, C6 atoms are colored in cyan, N atoms are colored in yellow. Bottom, right: snapshot of the equilibrated POPC bilayer. Vertical lines indicate the boundaries between the four regions defined in the text. (TIF) [file pone.0023187.s003.tif]
