# Supplementary material for: Permeation through the Cell Membrane of a Boron-Based β-Lactamase Inhibitor
Source: PLoS One. 2011 Aug 17;6(8):e23187. doi: 10.1371/journal.pone.0023187 (PMC3157353; doi:10.1371/journal.pone.0023187)
Supplement: Text S1 — (DOC) [file pone.0023187.s004.doc]

### Supporting Information.

### Molecular Simulations

The BZB structure was extracted from the X-ray crystallographic structure of the AmpC b-lactamase/BZB complex (PDB code 1C3B), solved at 2.25 Å resolution [1]. A 50Å x 50Å POPC [2] bilayer patch was built via the membrane plug-in of the VMD program suite [3] resulting in 60 1-palmitoyl-2-oleoyl-phosphatidylcholine (POPC) lipid molecules, 30 in each leaflet of the bilayer. A 50Å x 50Å x 80Å box encapsulating the membrane was filled with water molecules by applying the xleap routine of AMBER 8.0 [4] . The resulting system consisted of 16432 atoms.

All parameters of the POPC lipids except the charges were obtained using the Antechamber program [4], combined with the General Amber Force Field GAFF [5,6]. The RESP charges of POPC were calculated using GAUSSIAN03 at the Hartree-Fock 6-31G* level of theory on the optimized structure [7]. GAFF has been shown to provide a good description of POPC lipid properties [8,9]. We reproduce some of the previously calculated properties, thus ensuring the validity of our parameterization. The same procedure was applied to the parameterization of BZB, except for the B(OH)_2_ moiety for which the parameters of Tafi et al. [10] were used. The TIP3P model was used for water [11]. Periodic boundary conditions were applied. Long range electrostatic interactions were evaluated by the particle–Mesh Ewald (PME) algorithm using a 12 Å cut-off for the real part. The same cut-off was used for van der Waals interactions. The non-bonded atoms pair list was updated every 20 fs. The time-step was 1 fs. All simulations were performed in an isothermal-isobaric ensemble (1 atm, 300 K) with a Nosè–Hoover Langevin barostat (oscillation period 200 fs, decay coefficient 100 fs) and a Langevin thermostat (damping coefficient 1 ps).

MD run and analysis

The POPC model membrane was energy-minimized for 500,000 steps with steepest descent algorithm. Then the solvated membrane was equilibrated for 4.6 ns. The final size of the box was = 45x46x77 Å^3^. Several properties were calculated in the last 300 ps:

- **Area per lipid**. The area per lipid A was calculated dividing the lateral area of the simulation box by the number of lipid molecules in one leaflet. The calculated value was A = 69 ± 1 Å^2^, in agreement with the experimental result A = 68.3 ± 1.5 Å^2^ [8,9,11,12].
- **Lateral diffusion coefficient.** We used the Einstein diffusion equation to evaluate the lateral diffusion coefficient D: $D=\frac{\overline{\chi}^{2}}{2\Delta t}$ were χ is the mean square displacement and Dt is the size of one time step. The lateral diffusion coefficient D_lat_ was obtained from the slope of the curve of the mean square displacement versus time, calculated by the g_rmsd routine of the GROMACS package [9]. The calculated value was (0.07 ± 0.01) x10^-6^ cm^2^/s, consistently with experimental data and previous molecular dynamics simulations [8,9,12].
- **Structural facets.** The distances between phosphates on different leaflets of the bilayer (34.8 Å ± 0.3 Å), nitrogens (36.0 Å ± 0.3 Å) and C6 carbon atoms (27.7± 0.6 Å), see Fig. S3, agree with the mean distances reported in literature [12,13]. The distance between phosphate and nitrogen atoms within the same POPC molecule agrees with experimental data (model: P-N-dist = 4.5± 0.3 Å experimental data 4.3-4.5 Å for DOPC [14]).

BZB was then included in the proximity of the membrane surface. The system underwent additional 1 ns of MD. Then, metadynamics simulations were carried out. During the metadynamics runs, these properties remained rather close to the values above reported.

### Metadynamics.

Metadynamics consists in a continuous addition of a history dependent potential energy that enforces the dynamics to explore conformations that were not previously visited [15]. Briefly, the forces acting on each atom are corrected by a history dependent contribution, obtained as the derivative of the history dependent potential energy *G_t_* with respect to a chosen reaction coordinate. We chose the z-component of the distance vector joining the POPC membrane and the BZB center of mass (z-dist) as reaction coordinate. *G_t_* is given by the sum of a set of Gaussian functions centered on values *s_t_* of each chosen reaction coordinate (or metacoordinate) *s* at time *t*:

$$G_{t} =\sum_{\tau<t} we^{\left[-\frac{\left(s-s_{t}\right)^{2}}{2\delta^{2}}\right]}$$

The time interval between the addition of two Gaussian functions τ, as well as the Gaussian height w and Gaussian width δ, are tuned to optimize the ratio between accuracy and computational cost. Eventually, after exploring all conformations defined within the reaction coordinate space, the probability distribution of Gaussians becomes flat, and the free energy profile does not change any more. At this stage, the free energy surface can be easily reconstructed as the opposite of the sum of all Gaussians. We used: τ = 100 fs, w = 0.2 kJ/mol, δ = 0.5 Å

The free energy profile along the z-dist (G(z)) is shown in Fig. S3a, with the profile calculated every 1 ns up to 15 ns, with the last 400 ps smoothed. We identified the first well converged G(z) after 11 ns, then we obtained 5 independent free energy surfaces. The average <G(z)> and standard deviation were calculated over this set (Fig S3b) [16]. Given the high anisotropic behavior of lipids, the system was divided into four regions [17], symmetric with respect to the centre of the membrane (z=0) :1) a low head group density region at the water lipid interface (z>20); 2) a high head group density region, which corresponds to the membrane surface(12.5<z<20); 3) a high density region, at the beginning of the lipid tails (5.5<z<12.5); 4) a low density region, which corresponds to the center of the bilayer (0<z<5.5). *G(z)* in each region was estimated as the average of <G(z)> (red stars Fig. S3b).

Based on our metadynamics calculation, we calculated the permeability coefficient. Its inverse can be expressed as the integral over the local resistance across the membrane [18]:

$$\frac{1}{P}=\int_{0}^{d} \frac{dz}{K(z)D(z)}$$

*K (z)* is the partition coefficient from water into the membrane, while *D(z)* is the diffusion coefficient into the membrane at depth *z*. It is related to the free energy by the equation:

Assuming that the transport is governed by a distinct and uniform barrier region inside the membrane the equation can be simplified as [19,20]:

$$\frac{1}{P}=\frac{d}{KD}$$

Where *d* is the thickness of the barrier domain, *K* and *D* the partition coefficient and the diffusion coefficient through the barrier region, respectively.

## References

1. Powers RA, Blazquez J, Weston GS, Baquero F, Shoichet BK (1999) The complexed structure and antimicrobial activity of a non-β-lactam inhibitor of AmpC β-lactamase. Protein. Sci. **8**: 2330-2337.
2. Mac Callum JL, Tieleman DP (2008) Interactions between Small Molecules and Lipid Bilayers. *Current Topics in Membranes* **60**: 227-256.
3. Humphrey W, Dalke A, Schulten K (1996) VMD - visual molecular dynamics, *J. Mol. Graphics* **14**: 33–38.
4. Case DA, Cheatham TE, Darden T, Gohlke H, Luo R, et al. (2005) The Amber biomolecular simulation programs. *J Computat Chem* **26**: 1668-1688.
5. Ponder JW, Case DA (2003) Force Fields for Protein Simulations. *Adv. Prot. Chem* **66**: 27-85.
6. Cheatham TE, Youn MA (2001) Molecular dynamics simulation of nucleic acids: successes, limitations, and promise. *Biopolymers* **56**: 232-256.
7. Frisch MJ, Trucks GW, Schlegel HB, Scuseria GE, Robb MA, et al. (2004), Gaussian Inc, Wallingford CT.
8. Jojart B, Martinek TA (2007) Performance of the general amber force field in modeling aqueous POPC membrane bilayers. *J Comput Chem* **28**: 2051–2058.
9. Pantano S, Carafoli E (2007) The role of phosphorylation on the struscture and dynamics of phospholamban: A model from molecular simulations. *Proteins* **66**: 930-40.
10. Jorgensen WL, Chandrasekhar J, Madura JD, Impey RW, Klein ML (1983) Comparison of Simple Potential Functions for Simulating Liquid Water. *J Chem Phys* **79**: 926–935.
11. Kucerka N, Tristram-Nagle S, Nagle JF (2006) Structure of Fully Hydrated Fluid Phase Lipid Bilayers with Monounsaturated Chains, *J Membrane Biol* **208**: 193-202.
12. Tieleman DP, Marrink SJ, Berendsen HJC (1997) A computer perspective of membranes: molecular dynamics studies of lipid bilayer systems. *Biochim. Biophys. Acta* **1331**: 235–270.
13. Heller H, Schaefer M, Shulten K (1993) Molecular dynamics simulation of a bilayer of 200 lipids in the gel and in the liquid crystal-phases. J Phys Chem **97**: 8343-8360.
14. Bolterauer C, Heller H (1996) Calculation of IR dichroic values and order parameters from molecular dynamics simulation and their application to structure determination of lipid bilayers. *Eur J Biophys* **24**: 322–334.
15. Laio A, Parrinello M (2002) Escaping free-energy minima. *Proc Natl Acad Sci U S A* **99**: 12562–12566.
16. Micheletti C, Laio A, Parrinello M (2004) Reconstructing the Density of States by History-Dependent Metadynamics. Phys Rev Lett 92, 170601.
17. Marrink SJ, Berendsen HJC (1994) Simulation of water transport through a lipid membrane. *J Phys Chem* **98**:4155-4168.
18. Bemporand D, Luttmann C, Essex JW (2004) Computer Simulation of Small Molecule Permeation across a Lipid Bilayer: Dependence on Bilayer Properties and Solute Volume, Size, and Cross-Sectional Area. *Biophys. J*. **87**: 1-13.
19. Xiang TX, Anderson BD (1994) The relationship between permeant size and permeability in lipid bilayer membranes. *J* *Membr Biol* **140**: 111.
20. Xiang TX, Anderson BD (2000) Inﬂuence of a transmembrane protein on the permeability of small molecules across lipid membranes. *J Membr Bio* **173**:187–201.
